# Reliability and validity study of the Thai adaptation of the Copenhagen Burnout Inventory-Student Survey (CBI-SS) among preclinical medical students at the Faculty of Medicine Siriraj Hospital, Mahidol University, Thailand

**DOI:** 10.1371/journal.pone.0261887

**Published:** 2021-12-30

**Authors:** Wasit Wongtrakul, Yodying Dangprapai, Nattha Saisavoey, Naratip Sa-nguanpanich

**Affiliations:** 1 Department of Research and Development, Faculty of Medicine Siriraj Hospital, Mahidol University, Bangkok, Thailand; 2 Department of Physiology, Faculty of Medicine Siriraj Hospital, Mahidol University, Bangkok, Thailand; 3 Department of Psychiatry, Faculty of Medicine Siriraj Hospital, Mahidol University, Bangkok, Thailand; Medical University Innsbruck, AUSTRIA

## Abstract

Burnout syndrome is a syndrome of emotional exhaustion, professional efficacy and cynicism. A significant proportion of medical students reported having burnout syndrome during their training in medical education. Several tools including the Copenhagen Burnout Inventory-Student Survey (CBI-SS) are considered to be a valid measurement of burnout syndrome in medical students. This study aimed to translate, culturally adapt, and validate the CBI-SS for assessing burnout syndrome among preclinical medical students in Thailand. This study was conducted during February to March 2019 at the Faculty of Medicine Siriraj Hospital, Mahidol University (Bangkok, Thailand), which is Thailand’s largest and oldest medical school, and Thailand’s largest national tertiary referral center. After receiving formal permission to do so from the copyright owner, the original English language version of the CBI-SS was translated to Thai language using an internationally recommended and accepted forward-backward translation protocol. The Thai version of the CBI-SS (Thai CBI-SS) comprises 25 items, including 6 items for personal burnout, 7 items for study-related burnout, 6 items for colleague-related burnout, and 6 items for teacher-related burnout. Standardized Cronbach’s alpha coefficient was calculated to evaluate internal consistency reliability, and correlation coefficient was computed to determine test-retest reliability. A total of 414 preclinical medical students participated in this study. Due to sub-optimal factor weights (<0.50), items 6, 10 and 17 were excluded. The Cronbach’s alpha coefficients of the 22-item Thai CBI-SS for personal, study-related, colleague-related, and teacher-related burnout were 0.898, 0.896, 0.910 and 0.900 respectively. The correlation coefficients for test-retest reliability after three weeks were 0.820, 0.870, 0.821, and 0.787 for personal, study-related, colleague-related, and teacher-related burnout, respectively. Maximum likelihood analysis with oblimin rotation indicated four main components, and confirmatory factor analysis revealed good fit indices of the Thai CBI-SS. Confirmatory factor analysis showed good fit indices of CBI-SS domains (χ2/df = 2.39; CFI = 0.957; GFI = 0.909; RMSEA = 0.058; TLI = 0.949; and NFI = 0.928). The convergent validity analysis using the Average Variance Extracted (AVE) and the Composite Reliability (CR) was adequate for all dimensions (personal: AVE = 0.626, CR = 0.893; study-related: AVE = 0.601, CR = 0.899; colleague-related: AVE = 0.677, CR = 0.913; teacher-related: AVE = 0.606, CR = 0.900). The HTMT values for all variables are in the range from 0.315 to 0.833, confirming the discriminant validity. The Thai CBI-SS was found to be a valid and reliable tool for evaluating burnout syndrome in preclinical medical students in Thailand.

## Introduction

Burnout syndrome is a syndrome of emotional exhaustion that can manifest as any combination of the following: low mood, anxiety, irritability, and lack of professional efficacy, including poor motivation, procrastination, detachment from work, and having feelings of cynicism [1], that can result from long-term unresolved work-related stress [2]. Burnout showed clinical overlap with depression [3, 4], which is a serious psychiatric disorder that affects approximately 264 million people worldwide [5]. A significant proportion of medical students reported having burnout syndrome during their training in medical education [6, 7]. This finding aroused concern among faculty members of medical schools to identify risk factors, and to initiate proactive strategies to prevent burnout syndrome among medical students [8, 9]. Risk factors for burnout in medical students were reported to be male gender, lack of social support, and studying in more senior years [10].

The Maslach Burnout Inventory (MBI) was the most commonly used tool for assessing burnout in general population, and the Maslach Burnout Inventory Student Survey (MBI-SS), which is a 16-item adaptation of the MBI for students, was the most popular commercial measurement for burnout in medical students [11–13]. However, the MBI and MBI-SS were criticized for having limited conceptualization of burnout syndrome and for having poor psychometric properties. Depersonalization dimension reflected coping strategy rather than an essential part of the burnout syndrome. Moreover, personal accomplishment subscale of MBI was found to be weakly associated with other two subscales: the emotional exhaustion and depersonalization dimensions and might be only consequences of long-term stress [14, 15]. In response, researchers created several free-for-use alternative tools for evaluating burnout syndrome for scientific purposes, including the Copenhagen Burnout Inventory (CBI) [14] and Oldenburg Burnout Inventory (OLBI) [15]. The Copenhagen Burnout Inventory Student Survey (CBI-SS) is a student-specific adaptation of the CBI that comprises a total of twenty-five questions in four domains, including six items relating to personal burnout, seven items in study-related burnout, six items for colleague-related burnout, and six items concerning teacher-related burnout [16]. Although the CBI-SS has been linguistically translated and culturally adapted to many languages, it has not yet been adapted to Thai language and a Thai cultural setting. Accordingly, this study set forth to translate, adapt, and validate the CBI-SS for use in a Thai educational context. In addition to other educational settings, the Thai version of the CBI-SS (Thai CBI-SS) will improve our understanding of the prevalence and characteristics of burnout syndrome among preclinical medical students, which is a student subpopulation that is highly vulnerable to education-related burnout [17].

### Setting

Established in 1888, the Faculty of Medicine Siriraj Hospital, Mahidol University is the oldest and largest medical school in Thailand. Siriraj Hospital accepts approximately 80,000 inpatient cases and 3 million outpatient visits annually. An estimated 320 high school students are accepted annually into our 6-year undergraduate medical training program at the Faculty of Medicine Siriraj Hospital. Our curriculum includes a year of basic science, two years of preclinical medical courses, two years of clinical clerkship, and the last clerkship year as an extern. All medical graduates are expected to perform three years of medical internship in a government-sponsored hospital in a rural area before they can join a residency program.

## Methods

### Study design and study population

For exploratory factor analysis, 10–20 participants per question are usually required [18]. Therefore, all 322 second-year and 317 third-year preclinical medical students during academic year 2018–2019 were notified about this cross-sectional study to fulfil adequate sample size for exploratory factor analysis for twenty-five questions of CBI-SS. The participants were asked to consider voluntary participation as study subjects at the end of one of their mandatory classes during February-March 2019. Participating students accessed the Thai CBI-SS questionnaire via an online portal. Three weeks later [19], all students who had completed the Thai CBI-SS were asked to complete the Thai CBI-SS again via same online portal.

### Ethical considerations

Ethics approval for this study was granted by the Human Research Protection Unit of the Siriraj Institutional Review Board (SIRB) (Ethics ID 170/2561; EC1). Written informed consent was obtained from all study participants.

### Instruments

The authors obtained formal permission to translate the original version of the CBI-SS to Thai language from Prof. João Marôco, Instituto Superior de Psicologia Aplicada (ISPA), Lisbon, Portugal. The original version of the CBI-SS consists of a total of twenty-five questions in four domains, as follows: six items for personal burnout, seven items for study-related burnout, six items for colleague-related burnout, and six items for teacher-related burnout. Translation and cultural modification was based on a previously published study in the adaptation of the burnout inventory and World Health Organization guidelines [19, 20]. Forward translation from English to Thai was performed independently by a professional linguist from the Faculty of Liberal Arts, Mahidol University and an experienced psychologist from the Department of Psychiatry, Faculty of Medicine Siriraj Hospital, Mahidol University. Later, differences in translation were resolved by discussion and consensus between those two translators, which resulted in a final translation. That version was then reviewed by one of the authors (N.S.) who is well-acquainted with burnout syndrome. The agreed upon translation of the Thai CBI-SS was pretested in a pilot group of twenty medical students who neither demonstrated nor described any difficulty regarding the questions. Backward translation from Thai to English was then performed by a bilingual American professor from the translation unit of the Faculty of Arts, Chulalongkorn University, Bangkok, Thailand. The backward translated Thai CBI-SS displayed no major differences from the original version of the CBI-SS. The Thai version of the CBI-SS is shown in S1 Table.

### Statistical analysis

We used descriptive and analytical statistics to evaluate our data, and the mean scores of the Thai CBI-SS items were calculated.

All analytical techniques were based on those described in a previously published paper describing the translation and adaptation of the Serbian version of the MBI-SS [19]. Statistical Package for Social Science software (SPSS Inc, version 25, Chicago, IL, USA) was used for data analyses. Standardized Cronbach’s alpha coefficient was computed to evaluate internal consistency reliability, and correlation coefficient was calculated to determine test-retest reliability using intraclass correlation coefficient in participants who completed the Thai CBI-SS twice. The cut-off value for Cronbach’s alpha was 0.7. ICC > 0.75 was considered excellent, 0.4 to 0.75 as good and 0.4 < ICC as poor [21]. Fit indices examined were chi-square and degree of freedom ratio (χ2/df), comparative fit index (CFI), goodness of fit index (GFI), Tucker Lewis index (TLI), normed fit index (NFI) and root mean square error of approximation (RMSEA). When the RMSEA value less than 0.10, CFI, TLI, NFI, and GFI values greater than 0.90, the model indicates adequate fit [16]. A p-value of less than 0.05 was considered statistically significant for all tests. To determine the validity of the CBI-SS, we performed exploratory factor analysis and confirmatory factor analysis which examined goodness of fit index [22, 23], convergent validity using size of factor loading, the Average Variance Extracted (AVE) and the Composite Reliability (CR) [24, 25] and discriminant validity through HTMT [26]. The convergent validity and discriminant validity were considered adequate when AVE > 0.50, CR > 0.70 [27] and HTMT < 0.90 [26]. We also performed the maximum likelihood confirmatory factor analysis with oblimin rotation using the Analysis of Moment Structures (AMOS version 24.0) [28].

## Results

Of 639 eligible preclinical medical students, 414 students (64.8%) were enrolled in this study. Of those, 187 students (45.2%) took the Thai CBI-SS twice. Table 1 shows the demographic data of study participants (study year, gender, age distribution, hometown).

**Table 1:** Baseline demographic characteristics of study participants (N = 414).

Characteristics	n	%
Study year		
Year 2	216	52.2
Year 3	198	47.8
Gender		
Male	222	53.6
Female	192	46.4
Age (years)		
18	1	0.2
19	76	17.8
20	193	45.2
21	143	33.5
22	11	2.6
>22	3	0.6
Region of Thailand residence/origin		
Bangkok	231	55.8
Central	52	12.6
Northeastern	22	5.3
Northern	15	3.6
Southern	49	11.8
Eastern	13	3.1
Western	32	7.7

During preliminary confirmatory factor analysis, we found that the factor weights for item 6 (How often do you feel weak and susceptible to illness?), item 10 (Do you have enough energy for family and friends during leisure time?) and item 17 (Do you feel that you give more than you get back when you work with colleagues?) were 0.48, 0.36 and 0.36 respectively, all of which were considered to be sub-optimal and subsequently excluded. We continued the statistical analysis for 22 items. Table 2 demonstrates the Cronbach’s alpha coefficients of the 22-item Thai CBI-SS for personal burnout, study-related burnout, colleague-related burnout, and teacher-related burnout, which were 0.898, 0.896, 0.910 and 0.900 respectively. The overall Cronbach’s alpha coefficient of the Thai CBI-SS was 0.929. Table 3 shows excellent intraclass correlation coefficients for test-retest reliability 0.820, 0.870, 0.821, and 0.787 for personal burnout, study-related burnout, colleague-related burnout, and teacher-related burnout, respectively), which indicates good reliability of the Thai CBI-SS. Table 4 shows matrix of factor weights from exploratory factor analysis of CBI-SS items by oblimin rotation method.

**Table 2:** Internal consistency reliability of the Thai version of the CBI-SS in preclinical medical students at the Faculty of Medicine Siriraj Hospital, Mahidol University, Thailand.

Inventory			Mean	SD	Item-total correlation	Cronbach’s alpha coefficient
**CBI-SS**						0.929
	**Personal burnout**					0.898
		Item 1	3.58	0.89	0.789	
		Item 2	3.37	0.97	0.766	
		Item 3	3.42	1.01	0.701	
		Item 5	3.06	1.01	0.819	
		Item 7	3.13	1.09	0.680	
	**Study-related burnout**					0.896
		Item 4	2.14	1.04	0.649	
		Item 8	3.02	1.16	0.612	
		Item 9	2.75	1.02	0.710	
		Item 11	3.25	1.09	0.786	
		Item 12	2.78	1.09	0.800	
		Item 13	2.96	1.23	0.776	
	**Colleague-related burnout**					0.910
		Item 14	2.31	1.10	0.769	
		Item 15	2.18	1.07	0.789	
		Item 16	2.18	1.03	0.843	
		Item 18	2.11	1.04	0.785	
		Item 19	2.06	1.13	0.680	
	**Teacher-related burnout**					0.900
		Item 20	2.14	1.01	0.712	
		Item 21	1.92	0.90	0.777	
		Item 22	1.99	1.00	0.825	
		Item 23	1.71	0.92	0.538	
		Item 24	1.78	0.92	0.787	
		Item 25	1.79	0.94	0.743	

**Abbreviations:** CBI-SS, Copenhagen Burnout Inventory-Student Survey; SD, standard deviation.

**Table 3:** The test-retest reliability is presented in intraclass correlation coefficient of Thai CBI-SS.

Inventory	ICC	95%CI
Personal burnout	0.820	0.759–0.866
Study-related burnout	0.870	0.825–0.903
Colleague-related burnout	0.821	0.760–0.867
Teacher-related burnout	0.787	0.715–0.841

**Abbreviations:** CBI-SS, Copenhagen Burnout Inventory-Student Survey; ICC, Intraclass correlation coefficient; 95%CI: 95% confidence interval.

**Table 4:** Matrix of factor weights from exploratory factor analysis of CBI-SS items by oblimin rotation method.

Item	Factor 1	Factor 2	Factor 3	Factor 4
Item 1	0.855			
Item 2	0.851			
Item 3	0.739			
Item 5	0.848			
Item 7	0.700			
Item 4		0.669		
Item 8		0.622		
Item 9		0.708		
Item 11		0.870		
Item 12		0.875		
Item 13		0.844		
Item 14			0.822	
Item 15			0.844	
Item 16			0.905	
Item 18			0.821	
Item 19			0.713	
Item 20				0.767
Item 21				0.834
Item 22				0.885
Item 23				0.568
Item 24				0.827
Item 25				0.778

**Abbreviation:** CBI-SS, Copenhagen Burnout Inventory-Student Survey.

**Fig 1:**
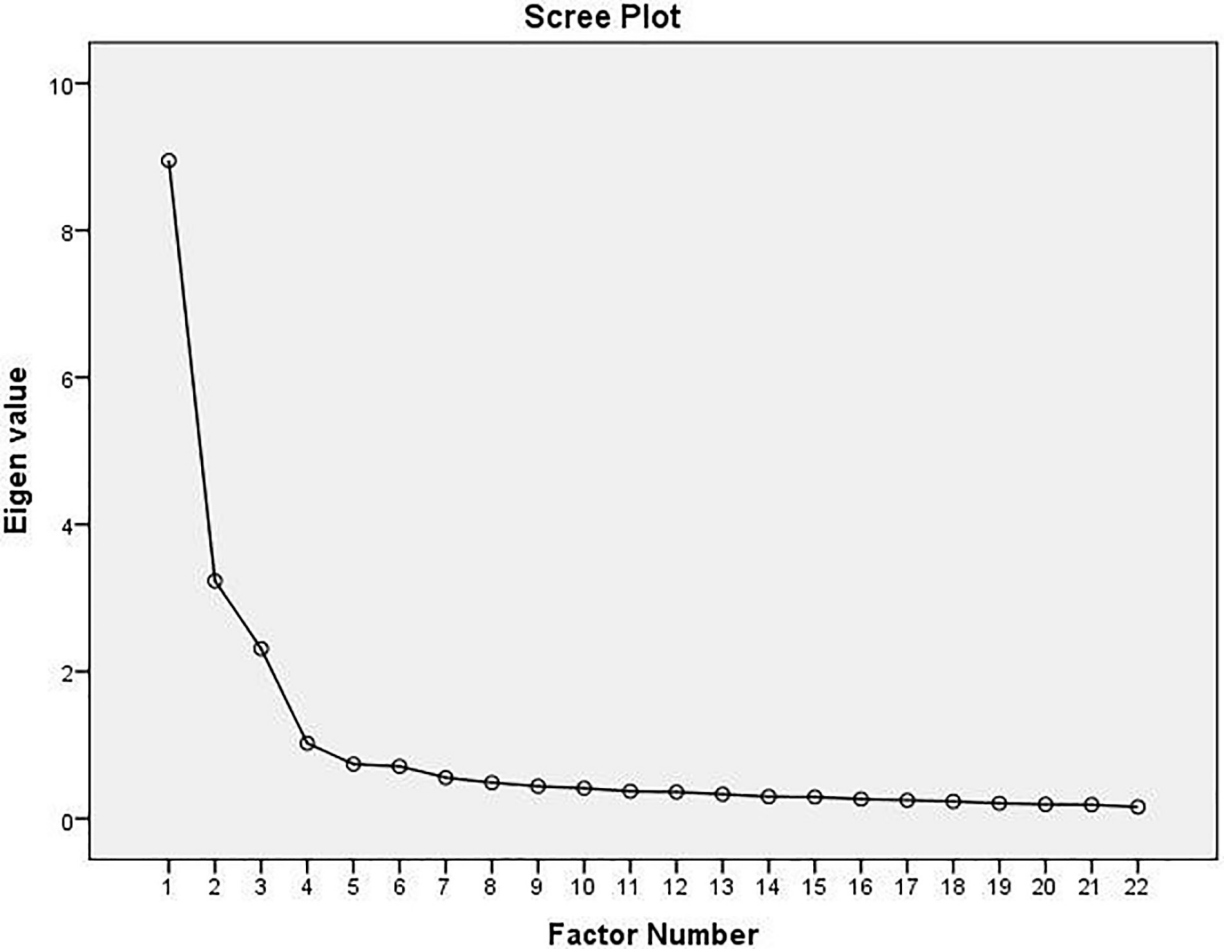
Screeplot of the components of the Thai version of the Copenhagen Burnout Inventory-Student Survey (CBI-SS).

**Fig 2:**
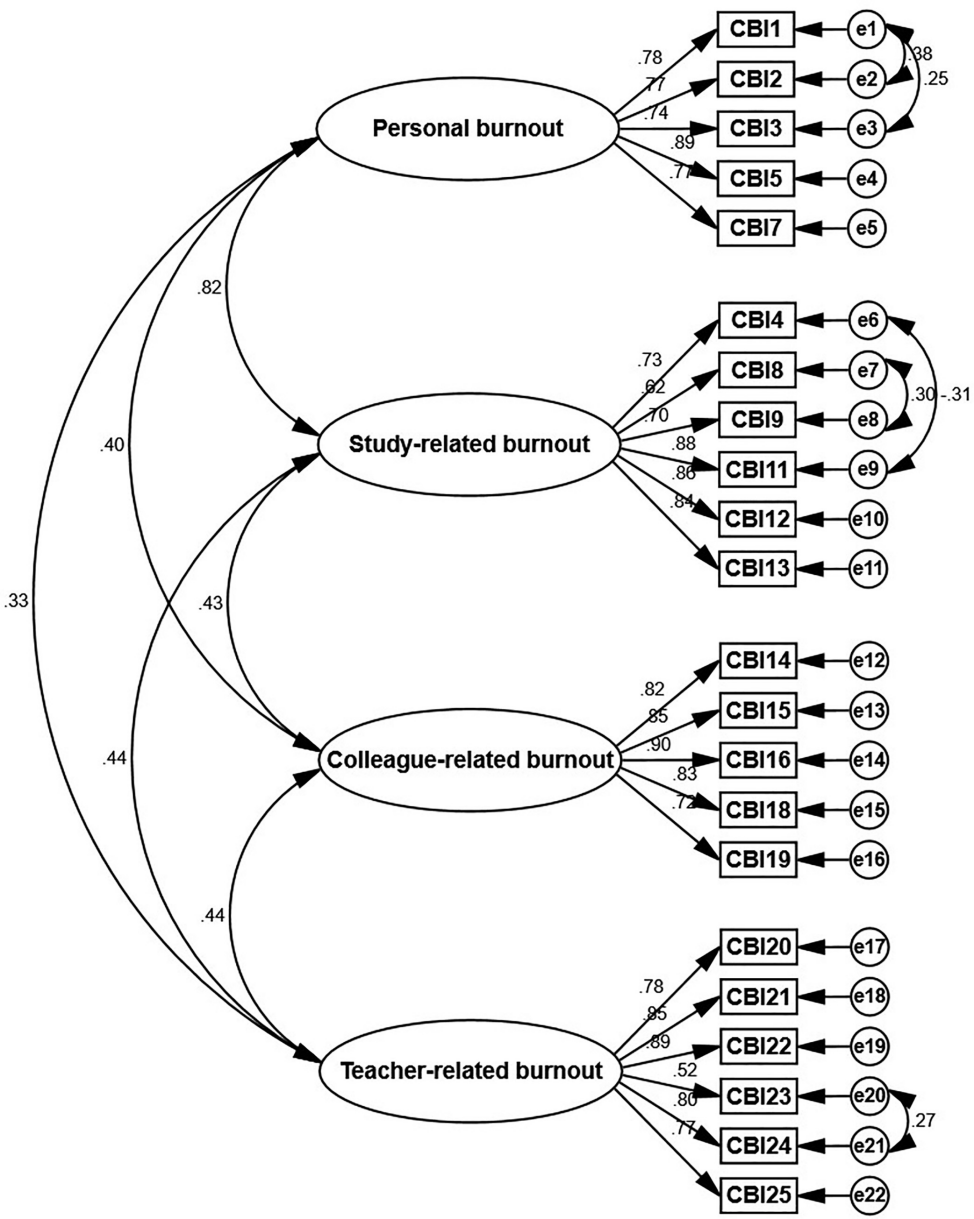
Confirmatory factor analysis of the Thai version of the Copenhagen Burnout Inventory (χ2/df =; CFI = 0.957; GFI = 0.909; RMSEA = 0.058; TLI = 0.949; and NFI = 0.928). (Abbreviations: χ2/df, chi-square and degree of freedom ratio; CFI, comparative fit index; GFI, goodness of fit index; NFI, normed fit index; TLI, Tucker Lewis index; RMSEA, root mean square error of approximation).

The result of maximum likelihood confirmatory factor analysis with oblimin rotation revealed the presence of four main factors with an eigenvalue greater than 1. Fig 1 shows a scree plot that supports a four-factor component. The path diagram in Fig 2 indicates that the standardized coefficients of the relationship between factors and items ranged from 0.52 to 0.90. Confirmatory factor analysis showed good fit indices of CBI-SS domains (χ2/df = 2.39; CFI = 0.957; GFI = 0.909; RMSEA = 0.058; TLI = 0.949; and NFI = 0.928). The convergent validity analysis using the AVE and the CR was adequate for all dimensions (personal: AVE = 0.626, CR = 0.893; study-related: AVE = 0.601, CR = 0.899; colleague-related: AVE = 0.677, CR = 0.913; teacher-related: AVE = 0.606, CR = 0.900). The HTMT values for all variables are in the range from 0.315 to 0.833, which are below 0.90, indicating acceptable values. Importantly, the result of HTMT infers that the variables are distinctively different from one another, which also confirms the discriminant validity.

## Discussion

After three items (6, 10, 17) were excluded, the results of this study showed excellent psychometric properties of the CBI-SS in Thai preclinical medical students. The Cronbach’s alpha coefficients of the CBI-SS were highly satisfactory, with values that range from 0.896 to 0.910 for all four subscales, and 0.929 for the total scale. However, our modified 22-item of Thai version CBI-SS yielded slightly lower Cronbach’s alpha coefficients values than the original Portuguese version of the CBI-SS whose values in subscales ranging from 0.875 to 0.931, and 0.957 for the total scale [16]. The test-retest correlation coefficients were within excellent range (approximately 0.787 to 0.870 for all subscales) despite the 3-week interval between the first and second Thai CBI-SS.

The only difference between the Thai CBI-SS and the original CBI-SS was that after exploratory factor analysis with oblimin rotation method with factor weight, we found that item 4 (How often do you think: “I can’t take it anymore”?) and 7 (“Do you feel worn out at the end of the working day?”) fit better in the study-related and personal burnout domain respectively instead of their original domain. This observation can be explained by linguistic difference during Thai translation. The Thai meaning of item 7 is more related to the personal inner self of respondents, which is why they were found to be a better fit with the personal burnout domain.

Portuguese version of CBI-SS showed factors of item 6 and 17 were 0.66 and 0.64 which were acceptable. However, its factor weight of item 10 was below 0.5, and item 10 was removed from Portuguese CBI-SS [16].

Consistent with the original version of the CBI-SS, the Thai CBI-SS was better fit with the 4-dimensional model with an eigenvalue greater than 1 [16].

### Strengths and limitations of the study

The strengths of this study include the large number of preclinical medical students that were enrolled from the largest medical school in Thailand, and the extensive statistical analyses that were employed to prove internal validity and reliability. The main limitation of this study was the lack of face-to-face evaluation of burnout syndrome after completion of the questionnaires.

## Conclusions

The 22-item Thai CBI-SS was found to be a valid and reliable tool for evaluating burnout syndrome in preclinical medical students in Thailand. The Thai CBI-SS and the data from this study will improve medical education research, our understanding of the characteristics and prevalence of burnout syndrome among Thai preclinical medical students, and will help us identify areas of improvement that will enhance the medical education process and experience.

## Supporting information

S1 Table Thai version of the Copenhagen Burnout Inventory Adapted for Students (CBI-SS). (PDF)
