# Supplementary material for: Reliability and validity study of the Thai adaptation of the Copenhagen Burnout Inventory-Student Survey (CBI-SS) among preclinical medical students at the Faculty of Medicine Siriraj Hospital, Mahidol University, Thailand
Source: PLoS One. 2021 Dec 30;16(12):e0261887. doi: 10.1371/journal.pone.0261887 (PMC8717990; doi:10.1371/journal.pone.0261887)
Supplement: S1 Table — (PDF) [file pone.0261887.s001.pdf]

**Supplementary Table 1.** Thai version of the Copenhagen Burnout Inventory Adapted for Students (CBI-SS)

| | | Never 0% of the time ไม่เคย | Rarely 25% of the time ไม่ค่อย | Sometimes 50% of the time บางครั้ง | Frequently 75% of the time บ่อย | Always 100% of the time เสมอ |
|---|---|---|---|---|---|---|
| | **Personal burnout** | | | | | |
| **1** | How often do you feel tired?<br>คุณรู้สึกเหนื่อยบ่อยแค่ไหน | 1 | 2 | 3 | 4 | 5 |
| **2** | How often are you physically exhausted?<br>มีคุณรู้สึกว่าร่างกายเหน็ดเหนื่อยอ่อนล้าบ่อยแค่ไหน | 1 | 2 | 3 | 4 | 5 |
| **3** | How often are you emotionally exhausted?<br>คุณรู้สึกว่าเหนื่อยใจบ่อยแค่ไหน | 1 | 2 | 3 | 4 | 5 |
| **4** | How often do you think: "I can't take it anymore"?<br>คุณคิดว่า "ทนไม่ไหวอีกแล้ว" บ่อยแค่ไหน | 1 | 2 | 3 | 4 | 5 |
| **5** | How often do you feel worn out?<br>คุณรู้สึกเหนื่อยล้าหมดแรงบ่อยแค่ไหน | 1 | 2 | 3 | 4 | 5 |
| **6** | How often do you feel weak and susceptible to illness?<br>คุณรู้สึกอ่อนแอและเจ็บป่วยได้ง่ายบ่อยแค่ไหน | 1 | 2 | 3 | 4 | 5 |
| | **Study-related burnout** | | | | | |
| **7** | Do you feel worn out at the end of the working day?<br>คุณรู้สึกหมดเรี่ยวแรงในวันทำงานใช่หรือไม่ | 1 | 2 | 3 | 4 | 5 |
| **8** | Are you exhausted in the morning at the thought of another day at work?<br>คุณรู้สึกเหนื่อยในตอนเช้าเมื่อคิดถึงงานที่ต้องทำในวันใหม่หรือไม่ | 1 | 2 | 3 | 4 | 5 |
| **9** | Do you feel that every working hour is tiring for you?<br>คุณรู้สึกว่าทุกชั่วโมงในการทำงานเป็นเรื่องน่าเหน็ดเหนื่อยหรือไม่ | 1 | 2 | 3 | 4 | 5 |
| **10*** | Do you have enough energy for family and friends during leisure time?<br>คุณมีพลังเหลือพอสำหรับครอบครัวและเพื่อนๆในช่วงเวลาพักผ่อนหรือไม่ | 1 | 2 | 3 | 4 | 5 |
| **11** | Are your studies emotionally exhausting?<br>การเรียนทำให้คุณรู้สึกเหนื่อยใจหรือไม่ | 1 | 2 | 3 | 4 | 5 |
| **12** | Do your studies frustrate you? | 1 | 2 | 3 | 4 | 5 |

| | | | | | | |
|---|---|---|---|---|---|---|
| | การเรียนทำให้คุณรู้สึกอึดอัดคับข้องใจหรือไม่ | | | | | |
| **13** | Do you feel burned out because of your studies?<br>คุณรู้สึกหมดไฟเพราะการเรียนของคุณหรือไม่ | 1 | 2 | 3 | 4 | 5 |
| | **Colleague-related burnout** | | | | | |
| **14** | Do you find it hard to work with colleagues?<br>คุณรู้สึกว่าทำงานกับเพื่อนร่วมชั้นเรียนได้ยากหรือไม่ | 1 | 2 | 3 | 4 | 5 |
| **15** | Does it drain your energy to work with colleagues?<br>การทำงานกับเพื่อนร่วมงานทำให้คุณหมดพลังหรือไม่ | 1 | 2 | 3 | 4 | 5 |
| **16** | Do you find it frustrating to work with colleagues?<br>คุณรู้สึกอึดอัดคับข้องใจเมื่อต้องทำงานกับเพื่อนร่วมชั้นเรียนหรือไม่ | 1 | 2 | 3 | 4 | 5 |
| **17** | Do you feel that you give more than you get back when you work with colleagues?<br>คุณรู้สึกว่าคุณต้องเป็นฝ่ายให้มากกว่ารับเมื่อทำงานกับเพื่อนร่วมชั้นเรียนหรือไม่ | 1 | 2 | 3 | 4 | 5 |
| **18** | Are you tired of working with colleagues?<br>คุณเบื่อที่จะทำงานกับเพื่อนร่วมชั้นเรียนหรือไม่ | 1 | 2 | 3 | 4 | 5 |
| **19** | Do you sometimes wonder how long you will be able to continue working with colleagues?<br>บางครั้งคุณก็สงสัยว่าคุณจะสามารถทำงานกับเพื่อนร่วมชั้นเรียนต่อไปไหวหรือไม่ | 1 | 2 | 3 | 4 | 5 |
| | **Teacher-related burnout** | | | | | |
| **20** | Do you find it hard to work with teachers?<br>คุณรู้สึกทำงานกับอาจารย์ได้ยากหรือไม่ | 1 | 2 | 3 | 4 | 5 |
| **21** | Does it drain your energy to work with teachers?<br>การทำงานกับอาจารย์ทำให้คุณหมดพลังหรือไม่ | 1 | 2 | 3 | 4 | 5 |
| **22** | Do you find it frustrating to work with teachers?<br>คุณรู้สึกอึดอัดคับข้องใจที่จะต้องทำงานกับอาจารย์หรือไม่ | 1 | 2 | 3 | 4 | 5 |
| **23** | Do you feel that you give more than you get back when you work with teachers? | 1 | 2 | 3 | 4 | 5 |

| | | | | | | |
|---|---|---|---|---|---|---|
| | คุณรู้สึกว่าคุณต้องเป็นฝ่ายให้มากกว่ารับเมื่อทำงานกับอาจารย์หรือไม่ | | | | | |
| **24** | Are you tired of working with teachers?<br>คุณเบื่อที่จะทำงานกับอาจารย์หรือไม่ | 1 | 2 | 3 | 4 | 5 |
| **25** | Do you sometimes wonder how long will be able to continue working with teachers?<br>บางครั้งคุณก็สงสัยว่าคุณจะสามารถทำงานกับอาจารย์ต่อไปไหวหรือไม่ | 1 | 2 | 3 | 4 | 5 |
